# Supplementary material for: Human iPSC-Derived Cortical Neurons Display Homeostatic Plasticity
Source: Life (Basel). 2022 Nov 14;12(11):1884. doi: 10.3390/life12111884 (PMC9696876; doi:10.3390/life12111884)
Supplement: Supplementary file 1 [file life-12-01884-s001.zip › life-2000422-supplementary.pdf]

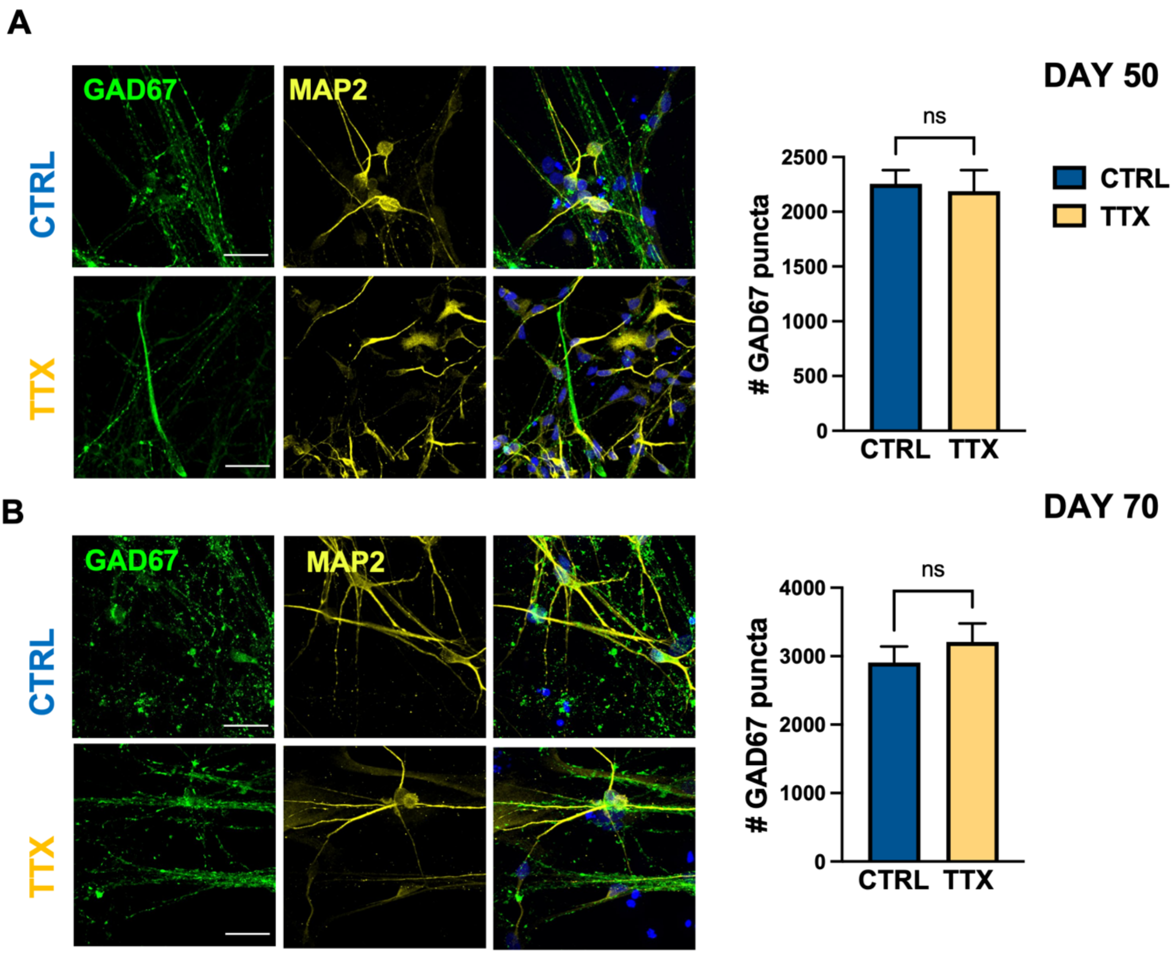

Supplementary Figure S1. TTX treatment does not alter the expression level of GABAergic presynaptic marker

Representative immunofluorescence images (left panels) of presynaptic GABAergic marker GAD67 in cortical cultures differentiated from hiPSCs in control conditions (CTRL; blue) and after 48h of TTX treatment (TTX; yellow) and relative bar chart for staining quantification (right panels). ***A.*** *GABAergic pre-synaptic puncta are identified as positive for GAD67 (green) at day 50, MAP2 (yellow) as neuronal marker (GAD67 puncta CTRL 2260 ± 130, TTX 2190 ± 190, p=0.76; t-test; n=28/2 FOVs/differentiation batches for each genotype).* ***B.*** *GABAergic pre-synaptic puncta are identified as positive for GAD67 (green) at day 70, MAP2 (yellow) as neuronal marker (GAD67 puncta CTRL 2910 ± 230, TTX 3210 ± 270, p=0.17; MW test; n=27/2 FOVs/differentiation batches for each genotype). Nuclei were stained with DAPI (blue); scale bar: 50 µm.*
